# Very Low Tidal Volume Ventilation with Associated Hypercapnia - Effects on Lung Injury in a Model for Acute Respiratory Distress Syndrome

**DOI:** 10.1371/journal.pone.0023816

**Published:** 2011-08-19

**Authors:** Hans Fuchs, Marc R. Mendler, Dominik Scharnbeck, Michael Ebsen, Helmut D. Hummler

**Affiliations:** 1 Division of Neonatology and Pediatric Critical Care, Department of Pediatrics and Adolescent Medicine, Ulm University, Ulm, Germany; 2 Medizinisches Versorgungszentrum Städtisches Krankenhaus Kiel, Kiel, Germany; Oregon Health and Science University, United States of America

## Abstract

**Background:**

Ventilation using low tidal volumes with permission of hypercapnia is recommended to protect the lung in acute respiratory distress syndrome. However, the most lung protective tidal volume in association with hypercapnia is unknown. The aim of this study was to assess the effects of different tidal volumes with associated hypercapnia on lung injury and gas exchange in a model for acute respiratory distress syndrome.

**Methodology/Principal Findings:**

In this randomized controlled experiment sixty-four surfactant-depleted rabbits were exposed to 6 hours of mechanical ventilation with the following targets: Group 1: tidal volume = 8–10 ml/kg/PaCO_2_ = 40 mm Hg; Group 2: tidal volume = 4–5 ml/kg/PaCO_2_ = 80 mm Hg; Group 3: tidal volume = 3–4 ml/kg/PaCO_2_ = 120 mm Hg; Group 4: tidal volume = 2–3 ml/kg/PaCO_2_ = 160 mm Hg. Decreased wet-dry weight ratios of the lungs, lower histological lung injury scores and higher PaO_2_ were found in all low tidal volume/hypercapnia groups (group 2, 3, 4) as compared to the group with conventional tidal volume/normocapnia (group 1). The reduction of the tidal volume below 4–5 ml/kg did not enhance lung protection. However, oxygenation and lung protection were maintained at extremely low tidal volumes in association with very severe hypercapnia and no adverse hemodynamic effects were observed with this strategy.

**Conclusion:**

Ventilation with low tidal volumes and associated hypercapnia was lung protective. A tidal volume below 4–5 ml/kg/PaCO_2_ 80 mm Hg with concomitant more severe hypercapnic acidosis did not increase lung protection in this surfactant deficiency model. However, even at extremely low tidal volumes in association with severe hypercapnia lung protection and oxygenation were maintained.

## Introduction

Mechanical ventilation is lifesaving in acute respiratory distress syndrome (ARDS), but contributes to lung injury as excessive transpulmonary pressure and alveolar strain lead to formation of alveolar edema, cytokine release from lung endothelial and epithelial cells and infiltration of the lung tissue with granulocytes [1], [2].

Lung protective ventilation strategies in pediatric ARDS include the use of low tidal volumes, avoidance of high peak inspiratory pressures and eventually permission of hypercapnia [3]. The use of low tidal volumes of 6 ml/kg as compared to high tidal volumes of 12 ml/kg decreased the mortality rate of ARDS in adults in the ARDS network trial [4]. There is less information on the effects of tidal volumes below 6 ml/kg on the degree of lung injury. Lower tidal volumes might be more lung protective [5]–[9] but ventilation with too low tidal volumes might increase lung injury by causing atelectasis, increased dead space ventilation, intrapulmonary shunting, hypoxia and hypercapnia [10]–[13].

An increased ventilation rate may allow only to compensate in part for the decrease in tidal ventilation resulting in hypercapnic acidosis [4], [7], [9]. There is some evidence from cell culture and animal studies including our own studies using this surfactant depletion model (unpublished data), that hypercapnic acidosis itself induced by administration of carbon dioxide to the inspired air (so called therapeutic hypercapnia) protects the lung [14]–[16] and increases oxygen delivery to the tissues [17]. Nevertheless, in clinical practice therapeutic hypercapnia has not emerged as a concept of therapy. Rather there is a controversy whether associated hypercapnic acidosis caused by hypoventilation should be “permitted” at all and which level of hypercapnia may be acceptable. In adults most recently high levels of hypercapnia associated with hypoventilation are lowered by pump less extracorporeal CO_2_ elimination [6], [7], [9], but so far this not an option for smaller children.

Clinically, in infants and children with severe ARDS over the past years we have accepted increasing levels of hypercapnia up to a maximum PCO_2_ of 120–140 mm Hg, if necessary to limit peak inspiratory pressures in order to avoid additional lung injury from ventilation. Tidal volumes in these patients often may be well below 5 ml/kg. This strategy is reserved only for the sickest pediatric ARDS patients and has been used especially in infants and children with respiratory failure after stem cell transplantation that do not qualify for extracorporeal membrane oxygenation therapy. So far, this strategy has to be considered as an experimental approach in individual patient care.

In this study we investigated this clinical scenario of down-titration of the tidal volumes in association with progressively high PaCO_2_ to evaluate the effects of this strategy on lung injury, gas exchange and on hemodynamics in a controlled established animal model for ARDS. We studied the hypothesis, that the reduction of tidal volume to allow for very severe hypercapnia would protect the lung in a “dose”-dependent fashion in this surfactant depletion model for ARDS. We further explored the effects of different hypercapnia levels on the cardiovascular system in this animal model.

## Methods

The experiments were approved by the Governmental *Animal Care Committee* at the Regierungspräsidium Tübingen, Baden-Württemberg, Germany (approval number: TVNr. 848) and were performed in accordance with current animal care guidelines.

### Animals

64 female adult New Zealand white rabbits weighing (2869+/−153 g) were obtained from Harlan GmbH, Borchen, Germany and housed standardized with 12∶12 h dark light circle with free access to water and food.

### Instrumentation and experimental procedure

After premedication with atropine 1 mg i.v., Ketamine 15–40 mg/kg i.v. and Xylazine 1.5–4 mg/kg i.v. animals were intubated orally with a 3.0 or 3.5 mm cuffed endotracheal tube. Thereafter, anesthesia was maintained by a continuous infusion of Ketamine (30–100 mg/kg/h) and Xylazine (0.3–1 mg/kg/h). The animals received 5 ml/kg/hr D5 (+70 mEq/L Na^+^, 18 mEq/L K^+^). During instrumentation animals were ventilated with the following settings: fraction of inspired oxygen: 0.4; tidal volume (V_T_): 7.5 ml/kg; positive end-expiratory pressure (PEEP): 4 cm H_2_O; inspiratory time: 0.5 sec; f = 20/minute. The rate was adjusted to obtain a PaCO_2_ in the target range (35–45 mm Hg). A Stephanie infant ventilator (Stephan, GmbH, Medizintechnik, Gackenbach, Germany) was used throughout the experiments using its calibrated high resolution pneumotachograph. A pulse oximeter (Radical, Masimo, Irvine, CA) was placed on a shaved foreleg. A 3F Pulsionkath thermodilution catheter (Pulsion Medical Systems, Munich, Germany) was introduced into the left femoral artery to measure blood pressure, cardiac output and to obtain arterial blood gases. A central venous catheter (3.5 Ch, Tyco, Tullamore Ireland) was introduced into the left femoral vein to measure central venous pressure. Cardiac output was measured by transpulmonary thermodilution by injection of 2 ml icecold normal saline into the central venous catheter using a PICCO plus (Pulsion Medical Systems, München, Germany). In the last 13 animals of this series a codman microsensor probe (Johnson and Johnson, New Brunswick, New Jersey) was inserted at 1 cm depth through a small borehole in the left frontoparietal bone to measure intracranial pressure.

Surfactant depletion was induced by repeated lung lavage at fraction of inspired oxygen 1.0 and PEEP 4 cm H_2_O with prewarmed normal saline in aliquots of 15 ml/kg. Lavages were repeated until the PaO_2_ was <200 mm Hg or <300 mm Hg with further continuous decrease of PaO_2_ as shown by subsequent arterial blood gas analyses q 5 minutes or until a total of 5 lavages. The lavage fluid (initial bronchoalveolar lavage, BAL) was analyzed for cell count (normalized for volume) and differential cell count. After lung lavage animals were randomized into 4 groups using sealed opaque envelopes.

According to the groups the animals were ventilated with the following settings and target ranges in the volume controlled mode. 1. Group 40: Vt = 8–10 ml/kg/PaCO_2_ = 40 mmHg, 2. Group 80: Vt = 4–5 ml/kg/PaCO_2_ = 80 mm Hg, 3. Group 120: Vt = 3–4 ml/kg/PaCO_2_ = 120 mm Hg and 4. Group 160: Vt = 2–3 ml/kg/PaCO_2_ = 160 mm Hg. From a pilot trial we had estimated that these tidal volumes would result in the target ranges of hypercapnia at ventilator rates in the same range. The PEEP was increased to 6 cm H_2_O, fraction of inspired oxygen was maintained at 1.0. The target PaCO_2_ range had to be reached within 1 h and was maintained by adjustments of the respiratory rate. Vercuronium (0.1 mg/kg) was given every 15 minutes to avoid spontaneous breathing. Arterial blood gas analysis, cooximetry and lactate were obtained every 15 minutes and analyzed with an automatic blood gas analyzer (Omni S, Roche; Mannheim, Germany). Central venous blood samples were drawn every hour. Cardiac output was determined every hour by transpulmonary thermodilution in triplicate and results were averaged.

Hemodynamics were supported by up to two volume challenges of 10 ml/kg of NaCl 0.9% i.v. given over 15 minutes whenever the systolic blood pressure dropped below 50 mm Hg and by dopamine infusion given in increments at a rate of 5 µg/kg/minute whenever volume challenges were not effective. Volume challenges were repeated after 2 h if hypotension persisted. Metabolic acidosis was corrected whenever the base deficit exceeded 10 mEq/L by infusion of 5 mEq NaHCO_3_.

### Lung preparation and histology

After 6 h surviving animals were sacrificed with a thiopental (50 mg) overdose. Immediately after death the ventilator was set to a continuous airway pressure at 10 cm H_2_O. The chest was opened, and the right lung was clamped and excised. The right lung was weighed (wet weight) and a BAL was performed using 7.5 ml/kg normal saline. Total protein was measured, the cell count was determined with a Neubauer cytometer and the differential cell count was analyzed by direct light microscopy after cytospin and Diff-Quick stain. Thereafter, the lung was dried for at least 7 days at 37°C and weighed daily until stable for at least 3 repeated measurements (dry weight). Before removal of the left lung the pulmonary artery was cannulated, the left atrium opened and the lungs vessels perfused with Ringers lactate solution preconditioned with 95% O_2_ and 5% CO_2_ and containing 2.2 mEq/L Ca^++^, 250 mg/l procaine and 20 U/ml heparin until clear fluid drained from the left atrium. Subsequently the lung was fixated by infusing 4.6% formaldehyde and 0.5% glutaralehyde for at least 10 min. Finally the trachea was ligated and the left lung was removed and submersed in fomaldehyde-glutaraldehyd solution at PEEP 10 cm H_2_O overnight. 4 specimens from anterior/posterior/upper/lower parts of the lung were embedded in paraffin and stained with hematoxylin-eosin. Lung injury was assessed by a pathologist blinded to the group assignment. To assess the degree of lung injury a previously described [18] modified score was assigned to each of the following nine variables: alveolar and interstitial inflammation, alveolar and interstitial hemorrhage, alveolar and interstitial edema, atelectasis, overinflation and necrosis. Each variable was analyzed at magnifications of 40x and 100x and scored using a 0–4 point scale. Zero represented no injury; 1, injury in 25% of the field; 2, injury in 50% of the field; 3, injury in 75% of the field; 4, injury in 100% of the field. A sum score was calculated and thus the maximum possible score was 144.

### Data analysis and statistics

For continuous real time data acquisition Windaq Pro+ (DataQ, Acron, OH) was used. Data were processed in Excel (Microsoft, Redmond, WA) and analysed using SigmaStat V2.03 (Systat Software, San Jose, California). Animals surviving until the end of the experiments were included into analysis per protocol. The sample size calculation was based on the following considerations: The primary outcome was the wet-dry weight ratio of the right lung. According to our previous studies we expected a mean wet dry weight ratio of 12.01 (expected SD 2.89). To correct for multiple comparisons a Bonferroni correction of the alpha error was done to allow for the 6 possible comparisons between the four groups: (alpha 0.05/6 = 0.0083). 15 animals per group would be necessary to detect a 30% reduction in the wet-dry weight ratio measurements (expected Δ = 3.61) with α = 0.05 and β = 0.2 (ANOVA).

Data were analyzed using ANOVA, ANOVA on ranks and repeated measures ANOVA. Parameters of interest are given averaged over experimental time (0.5–6 h). Mean (standard deviations) or median (interquartile ranges) are given as appropriate. A p-value <0.05 was considered significant.

## Results

### Mortality

Two animals died before the end of the experimental time in group 40 due to pneumothorax, one animal died in group 120 due to iatrogenic air embolism, one animal died in group 160 due to acute severe hypoxemia. According to protocol these animals were excluded from further analysis. However, inclusion of these animals would not have had an impact on any results.

### Wet-dry weight ratio of the right lungs, lung mechanics

**Figure 1:**
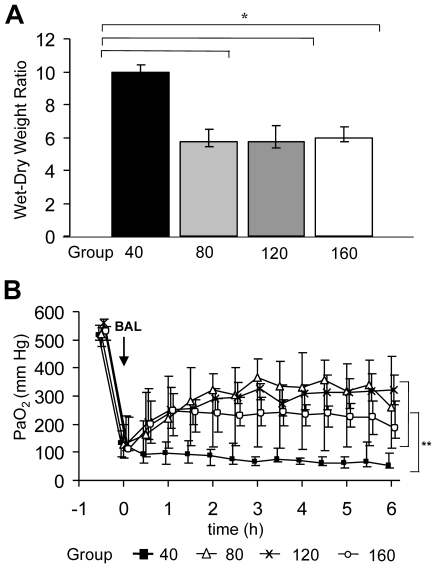
Markers of lung injury. The wet-dry weight ratio of the right lung (A). PaO_2_ across time (B). Median and interquartile ranges are shown. *p<0.001 (ANOVA); **p<0.01 (repeated measures ANOVA on ranks).

All three hypercapnia groups had lower wet-dry weight ratios of the right lung as compared to group 40 (p<0.001; Fig. 1A). The wet dry weight ratio was similar in group 80, group 120 and group 160 (Fig. 1A). Data on lung mechanics are given in Table 1.

**Table 1:** Blood gases, respiratory mechanics and intracranial pressure.

Group	40	80	120	160	
pH	7.29 (7.27–7.3)	7.09 (7.08–7.12)	6.98 (6.97–7.0)	6.91(6.88–6.92)	p<0.001*
PaCO_2_ (mm Hg)	40.3 (39.9–42.3)	80.3 (79.2–83.5)	120.6 (116.6–123.7)	156.8 (154.0–161.6)	p<0.001*
Base deficit (mEq/L)	7.1 (6.1–8.1)	7.9 (5.5–8.5)	7.1 (6.8–7.7)	7.5 (6.2–8.7)	n.s.
Lactate (mEq/L)	2.8 (1.7–4.0)	0.9 (0.6–1.5)	0.7 (0.6–0.9)	1.7 (1.1–2.6)	p<0.05^†^
Paw (cm H_2_O)	9.3 (7.9–10.2)	7.4 (7.1–8.0)	6.9 (6.7–7.3)	6.8 (6.8–7.2)	p<0.05**
PIP (cm H_2_O)	18.2 (16.5–20.3)	10.0 (8.3–11.9)	8.6 (7.3–9.7)	8.0 (6.7–9.7)	p<0.01^‡^
Ventilator rate (breath/min)	26 (23–34)	26 (24–35)	22 (19–33)	23 (21–34)	n.s.
Dynamic compliance (ml/cmH_2_O/kg)	0.68 (0.59–0.75)	0.56 (0.45–0.74)	0.53 (0.5–0.62)	0.56 (0.48–0.76)	n.s.
ICP (cm H_2_O)	7.6 (7.1–8.6)	5.8 (5.5–6.8)	5.8 (4.8–6.4)	4.2 (3.8–6.8)	–

Median (interquartile ranges); Paw = mean airway pressure, PIP = peak inspiratory pressure, ICP = intracranial pressure.

* = p<0.001: 40 vs 80 vs 120 vs 160; ** = p<0.05: 40 vs 80, 80 vs 120; p<0.005: 40 vs 120, 40 vs 160; ^†^ = p<0.001: 40 vs 80, 40 vs 120; p<0.05: 40 vs 160, 120 vs 160; ^‡^ = p<0.01: 40 vs. 80, 40 vs 120, 40 vs 160; n.s.  =  not significant.

### Gas exchange, lactate

The target PaCO_2_ was reached within one hour and maintained throughout the experiment in all groups (Table 1). The increase in PaCO_2_ resulted in different degrees of acidosis (p<0.001; Table 1). All three hypercapnia groups had higher PaO_2_-levels during experimental time as compared to group 40 (p<0.01 Fig. 1B). PaO_2_ levels were similar in group 80, group 120 and group 160 (Fig. 1B). Lactate was higher in group 40 than in group 80 and 120 and 160 (p<0.05; Table 1).

### Lung histology

Less alveolar and interstitial inflammation of the lung were seen in group 80, group 120 and group 160 as compared to group 40 (p<0.001; Table 2). Furthermore, there was less alveolar and interstitial edema and atelectasis present in all hypercapnia groups as compared to group 40 (p<0.001; Table 2). The median sum score in group 40 was higher than in the hypercapnia groups (p<0.001). Lung injury as judged by histology was similar in group 80, group 120 and group 160 (Table 2).

**Table 2:** Lung histology scores.

Group	40	80	120	160	
Alveolar inflammation	5 (4–6)	0 (0–0.5)	0 (0–0)	0 (0–0)	p<0.001*
Alveolar hemorrhage	0 (0–2.5)	0 (0–0)	0 (0–1)	0 (0–0)	n.s.
Interstitial inflammation	5 (4–7)	0 (0–0.5)	0 (0–0)	0 (0–1)	p<0.001*
Interstitial hemorrhage	4 (3–5)	4 (2.5–6)	4 (2–10)	6 (5–10)	n.s.
Alveolar edema	4 (3–5)	0 (0–1)	0 (0–0)	0 (0–0)	p<0.001*
Interstitial edema	5 (2.5–7.5)	1 (0–3.5)	1 (0–2)	0 (0–1)	p<0.001*
Atelectasis	3 (1.5–4.5)	0 (0–0)	0 (0–0)	0 (0–0)	p<0.001*
Overdistension	10 (9–10.5)	11 (10–12)	11 (10–11.5)	10 (8–11.5)	n.s.
Necrosis	0 (0–0)	0 (0–0)	0 (0–0)	0 (0–0)	n.s.
Sum	45 (37–55)	20 (15–24)	19 (17–23)	21 (18–23)	p<0.001*

Median (interquartile ranges); * = 40 vs 80, 40 vs 120, 40 vs 160; n.s.  =  not significant.

### Bronchoalveolar lavage

**Figure 2:**
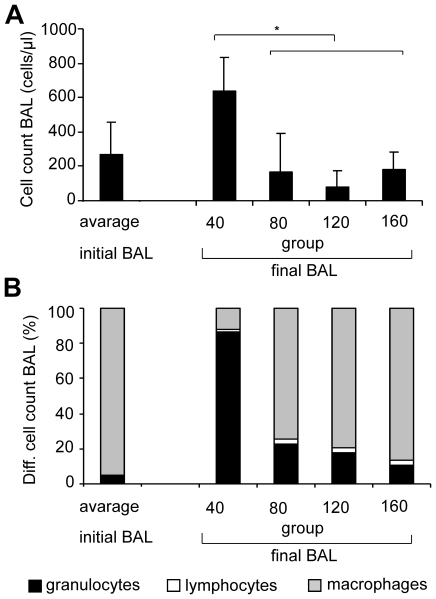
Bronchoalveolar lavage (BAL). Cell count (A) and differential cell count (B) were measured in the bronchoalveolar lavage fluid prior to intervention (initial BAL) and at the end of the experiment (final BAL). Data are averaged for the initial BAL and specified for the final BAL for group 40, group 80, group 120 and group 160. Median and interquartile ranges are shown. * = p<0.05 (ANOVA on ranks).

The inital BAL was performed to deplete surfactant prior to the intervention and a final BAL was performed post mortem in the excised right lung. There were no differences in protein contents or cell counts between groups in the initial BAL (Fig. 2). In the final BAL the median protein content was higher in group 40∶760 (419; 1030) mg/l as compared to group 80∶300 (180; 414) mg/l (p<0.05). It was 182 (79; 764) mg/l in group 120 and 585 (272; 758) mg/l in group 160. Data on the cellular contents of the BAL fluid are shown in Figure 2.

### Hemodynamics and interventions to treat hypotension and metabolic acidosis, intracranial pressure

**Figure 3:**
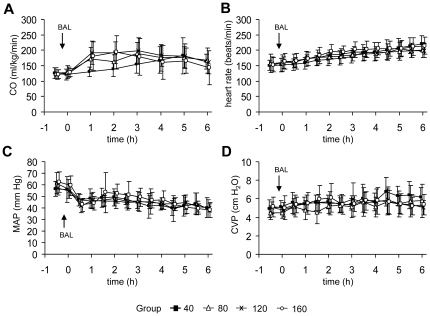
Hemodynamics. Cardiac output (CO, A), heart rate (B), mean arterial blood pressure (MAP, C), central venous pressure (CVP, D) across experimental time. Mean +/− SD are shown. There are no differences between groups (repeated measures ANOVA).

Heart rate, mean arterial blood pressure, central venous pressure and cardiac output were similar in the four groups throughout experimental time (Fig. 3). More cumulative additional Ringer fluid replacement was necessary according to protocol in group 40 compared to group 120 and group 160 [p<0.05; group 40∶30.6 (20.9–42.5) ml/kg; group 80∶20.1 (9.9–36.7) ml/kg; group 120∶20.0 (10.2–21.2); group 160∶10.5 (4.9–26.9)]. The average dopamine dose given according to the protocol was similar between groups. Cumulative NaHCO_3_ given was similar between groups [n.s.; group 40∶3.31 (1.7–4.3) ml/kg; group 80∶1.82 (0.85–3.48) ml/kg; group 120∶3.19 (1.71–3.47) ml/kg, group 160∶3.29 (1.69–3.67) ml/kg]. Intracranial pressure was recorded in 13 animals. Intracranial pressure was not elevated in the hypercapnia groups (Table 1).

## Discussion

We used a rabbit model of ARDS to explore the effects of a very low tidal volume ventilation strategy associated with very high levels of concomitant hypercapnia to mimic a strategy used in our pediatric intensive care unit to treat children with severe ARDS. We had hypothesized that a further reduction of the tidal volume below 4–5 ml/kg would result in increased lung protection in a dose dependent fashion. Similar to the clinical scenario accompanying extreme hypercapnia was allowed to eventually enhance lung protective effects of the low volume strategy or to assess potentially adverse effects of very severe hypercapnia to define an optimal range.

The wet-dry weight ratio is a marker for alveolar and interstitial edema formation, which is an early hallmark in the pathogenesis of lung injury. Lung micro vascular permeability has been shown to increase rapidly after injurious ventilation triggered by rapid signal transduction events involving stress pathway and cytokine activation [1], [2]. In our surfactant depletion model the use of tidal volumes in the range of 4–5 ml/kg associated with moderate hypercapnia protected from alveolar edema formation as compared to the accepted and commonly applied tidal volumes of 8–10 ml/kg [11], [19] with normocapnia. This was associated with decreased inflammation of the lung, as evidenced by histology and BAL cell count. Oxygenation was improved and lactate levels were decreased in all hypercapnia groups. However, we were not able to demonstrate additional benefit, when tidal volumes below 4–5 ml/kg associated with more severe hypercapnia were used. Therefore, no dose dependency was observed.

One possible explanation might be a threshold effect of tidal volume similar to that found for peak airway pressure [20]. Lung protection may be maintained as long as overdistension of the lungs by very large tidal volumes is avoided, but the use of very low tidal volumes does not add any additional benefits in this model.

However, in clinical practice, extremely low tidal volumes would be only applied, if moderate tidal volumes would result in too high peak inspiratory pressures. Our surfactant depletion model did not result in such a severe lung injury. In this study moderate low tidal volumes and moderate hypercapnia prevented injurious ventilation pressures and largely abolished lung injury, leaving little scope tracking differences between groups. Therefore, we can not rule out, that benefits from an extremely low tidal volume strategy might have become apparent in a setting with more severe initial lung injury.

Nevertheless, our data illustrate that lung protection is maintained at least equally if extremely low tidal volumes and severe hypercapnia are applied. In contrast to experimental [10] and clinical [12], [19] observations the use of lower edge tidal volumes was almost not associated with adverse gas exchange due to atelectasis, inhomogeneous air distribution or increased inflammation contradicting an U-shaped model for tidal volume and lung protection [19].

Functional residual capacity seems crucial to maintain lung protection at low tidal volumes [8], [21]. Worsening lung function secondary to hypoventilation in association with hypercapnia has been described in an animal model with endotoxic shock [22]. In that study, the use of very low PEEP (2 cm H_2_O) combined with a low rate might have allowed for more severe atelectotrauma and thus increased the degree of lung injury [22]. In contrast the PEEP applied in our study (6 cm H_2_O) may have been high enough to maintain sufficient residual capacity as evidenced by histology despite very small tidal volumes of 2–3 ml/kg.

In addition, in our study the severe hypercapnic acidosis might have enhanced hypoxic pulmonary vasoconstriction resulting in improved ventilation-perfusion matching which may at least in part compensate for oxygenation problems associated with an extremely low tidal volume strategy as suggested by several studies [23]–[25]. This might explain, why more hypoxemia due to derecruitment and intrapulmonary shunting was observed in a study in surfactant depleted swine at similar PEEP (5 cm H_2_O) compared to our study. In that study the use of low tidal volumes (3 ml/kg) was combined with extracorporeal CO_2_ elimination and normocapnia [10]. However, beneficial effects of hypercapnia on ventilation-perfusion matching may be limited to therapeutic hypercapnia [8], [12] and these measurements were not addressed by our study.

Antiinflammatory properties of the severe hypercapnic acidosis might attenuate lung injury in addition. We found a decrease in numbers of granulocytes in the BAL in a dose-dependent fashion in proportion to the degree of hypercapnia, suggesting that this effect might be linked directly to the expected anti-inflammatory properties of hypercapnic acidosis [26]. Inhibition of the activation of the key transcription factor of cell injury nuclear factor kappa B by hypercapnic acidosis was described before resulting in lower extravasations of polymorphonuclear cells via the reduced expression of intercellular adhesion molecule on lung endothelial cells [26].

Our experiments do not differentiate the effects of the low tidal volumes from the effects of hypercapnic acidosis. The design of this study was chosen to mimic the typical clinical scenario where hypercapnia has to be permitted in order to further decrease tidal volumes with the aim to explore if such a strategy results in an increased protection of the lung. We speculate that in this study low tidal volumes and hypercapnic acidosis have additive lung protective effects. However, the possibility that benefits of using a low tidal volume was counteracted by deleterious effects of the hypercapnic acidosis or that adverse effects of a too low tidal volume are attenuated by hypercapnia can not be ruled out by this study. Nevertheless, we have shown that the use of very low tidal volumes in combination with hypercapnia do not seem to harm. Further studies are needed to differentiate mechanisms involved in the pathogenesis of lung injury during hypercapnia.

There are several limitations of the surfactant deficiency model for ARDS. First, the initial phase of alveolar edema, inflammation and necrosis is only the first phase in the pathogenesis of permanent lung injury. It is followed by a phase of healing frequently accompanied by different degrees of fibrosis. Some studies suggest that hypercapnia might impair lung healing [27], [28]. Long term studies on hypercapnia in spontaneous breathing animals show conflicting results [29], [30]. From this six hour protocol we cannot extrapolate on events later in the course of ventilation-induced lung injury. However, even if there might be some adverse effects of hypercapnia compared to normocapnia on the pathogenesis of permanent lung injury at a later stage, obviously the price for maintenance of normocapnia would have been severe initial lung damage. Further long-term studies are needed to understand the influence of hypercapnia on later stages of ventilation-induced lung damage and recovery. Second, ARDS is often accompanied by bacterial infection [31], which is not present in our model. It has been shown, that hypercapnic acidosis may attenuate an inflammatory response which might impair the defence against microbes in prolonged bacterial infection by mechanisms involving inhibition of neutrophil phagocytosis [32]. However, in a model of severe pneumonia moderate hypercapnic acidosis demonstrated attenuation but not worsening of lung injury [33]. In addition, moderate hypercapnic acidosis did not alter lung or systemic bacterial loads in a model of prolonged systemic sepsis [34]. A clinical impact of a partially impaired host defence is questionable anyway, as patients usually are treated with broad spectrum antibiotics.

One major concern with hypercapnia is the adverse effect of acidosis on cardiac contractility. Clinically, the acute onset of moderate hypercapnia in ARDS patients seems to be well tolerated resulting in an increased cardiac index and oxygen delivery despite some negative effects on myocardial contractility [35]. The results of our and other experimental studies [17], [24] are reassuring, as even acute extreme hypercapnic acidosis was tolerated well by the animals without profound negative hemodynamic effects. However, these observations may be different in a setting with pre-existing cardiovascular illness. In our study less hemodynamic support, i.e. fluid replacement was necessary in all groups with hypercapnic acidosis and animals of hypercapnia groups did not require more inotropic support. In addition, we measured intracranial pressure in a subgroup of the animals as brain edema might be associated with acute hypercapnia [36]. However, no increased intracranial pressure was found in association with severe hypercapnia during experimental time in this animal model.

In conclusion, in comparison to tidal volumes of 8–10 ml/kg and normocapnia the use of low tidal volumes in combination with associated hypercapnia is capable to protect the lung, but not in a dose depended fashion. The reduction of the tidal volume below 4–5 ml/kg along with acceptance of more severe hypercapnic acidosis did not further enhance lung protection in this surfactant depletion model nor did it cause harm. No clinically relevant short term adverse effects, especially on gas exchange or hemodynamics were observed with decreasing tidal volume and increasing PaCO_2_. Clinical studies are warranted, to explore the effects of a very low tidal volume strategy with concomitant hypercapnia on long term outcome.

## References

[1] Yoshikawa S, King JA, Lausch RN, Penton AM, Eyal FG (2004). Acute ventilator-induced vascular permeability and cytokine responses in isolated and in situ mouse lungs.. J Appl Physiol.

[2] Held HD, Boettcher S, Hamann L, Uhlig S (2001). Ventilation-induced chemokine and cytokine release is associated with activation of nuclear factor-kappaB and is blocked by steroids.. Am J Respir Crit Care Med.

[3] Randolph AG (2009). Management of acute lung injury and acute respiratory distress syndrome in children.. Crit Care Med.

[4] The Acute Respiratory Distress Syndrome Network (2000). Ventilation with lower tidal volumes as compared with traditional tidal volumes for acute lung injury and the acute respiratory distress syndrome.. N Engl J Med.

[5] Terragni PP, Del Sorbo L, Mascia L, Urbino R, Martin EL (2009). Tidal volume lower than 6 ml/kg enhances lung protection: role of extracorporeal carbon dioxide removal.. Anesthesiology.

[6] Zimmermann M, Bein T, Arlt M, Philipp A, Rupprecht L (2009). Pumpless extracorporeal interventional lung assist in patients with acute respiratory distress syndrome: a prospective pilot study.. Crit Care.

[7] Muellenbach RM, Kredel M, Wunder C, Kustermann J, Wurmb T (2008). Arteriovenous extracorporeal lung assist as integral part of a multimodal treatment concept: a retrospective analysis of 22 patients with ARDS refractory to standard care.. Eur J Anaesthesiol.

[8] Muellenbach RM, Kredel M, Kuestermann J, Klingelhoefer M, Schuster F (2009). Combining “open-lung” ventilation and arteriovenous extracorporeal lung assist: influence of different tidal volumes on gas exchange in experimental lung failure.. Med Sci Monit.

[9] Bein T, Zimmermann M, Hergeth K, Ramming M, Rupprecht L (2009). Pumpless extracorporeal removal of carbon dioxide combined with ventilation using low tidal volume and high positive end-expiratory pressure in a patient with severe acute respiratory distress syndrome.. Anaesthesia.

[10] Dembinski R, Hochhausen N, Terbeck S, Uhlig S, Dassow C (2007). Pumpless extracorporeal lung assist for protective mechanical ventilation in experimental lung injury.. Crit Care Med.

[11] Ricard JD (2003). Are we really reducing tidal volume–and should we?. Am J Respir Crit Care Med.

[12] Feihl F, Eckert P, Brimioulle S, Jacobs O, Schaller MD (2000). Permissive hypercapnia impairs pulmonary gas exchange in the acute respiratory distress syndrome.. Am J Respir Crit Care Med.

[13] Pfeiffer B, Hachenberg T, Wendt M, Marshall B (2002). Mechanical ventilation with permissive hypercapnia increases intrapulmonary shunt in septic and nonseptic patients with acute respiratory distress syndrome.. Crit Care Med.

[14] Laffey JG, Honan D, Hopkins N, Hyvelin JM, Boylan JF (2004). Hypercapnic acidosis attenuates endotoxin-induced acute lung injury.. Am J Respir Crit Care Med.

[15] Sinclair SE, Kregenow DA, Lamm WJ, Starr IR, Chi EY (2002). Hypercapnic acidosis is protective in an in vivo model of ventilator-induced lung injury.. Am J Respir Crit Care Med.

[16] Broccard AF, Hotchkiss JR, Vannay C, Markert M, Sauty A (2001). Protective effects of hypercapnic acidosis on ventilator-induced lung injury.. Am J Respir Crit Care Med.

[17] Komori M, Takada K, Tomizawa Y, Nishiyama K, Kawamata M (2007). Permissive range of hypercapnia for improved peripheral microcirculation and cardiac output in rabbits.. Crit Care Med.

[18] Hummler HD, Thome U, Schulze A, Schnabel R, Pohlandt F (2001). Spontaneous breathing during partial liquid ventilation in animals with meconium aspiration.. Pediatr Res.

[19] Eichacker PQ, Gerstenberger EP, Banks SM, Cui X, Natanson C (2002). Meta-analysis of acute lung injury and acute respiratory distress syndrome trials testing low tidal volumes.. Am J Respir Crit Care Med.

[20] Parker JC, Townsley MI, Rippe B, Taylor AE, Thigpen J (1984). Increased microvascular permeability in dog lungs due to high peak airway pressures.. J Appl Physiol.

[21] Muscedere JG, Mullen JB, Gan K, Slutsky AS (1994). Tidal ventilation at low airway pressures can augment lung injury.. Am J Respir Crit Care Med.

[22] Lang JD, Figueroa M, Sanders KD, Aslan M, Liu Y (2005). Hypercapnia via reduced rate and tidal volume contributes to lipopolysaccharide-induced lung injury.. Am J Respir Crit Care Med.

[23] Swenson ER, Robertson HT, Hlastala MP (1994). Effects of inspired carbon dioxide on ventilation-perfusion matching in normoxia, hypoxia, and hyperoxia.. Am J Respir Crit Care Med.

[24] Wang Z, Su F, Bruhn A, Yang X, Vincent JL (2008). Acute hypercapnia improves indices of tissue oxygenation more than dobutamine in septic shock.. Am J Respir Crit Care Med.

[25] Ketabchi F, Egemnazarov B, Schermuly RT, Ghofrani HA, Seeger W (2009). Effects of hypercapnia with and without acidosis on hypoxic pulmonary vasoconstriction.. Am J Physiol Lung Cell Mol Physiol.

[26] Takeshita K, Suzuki Y, Nishio K, Takeuchi O, Toda K (2003). Hypercapnic acidosis attenuates endotoxin-induced nuclear factor-[kappa] B activation.. Am J Respir Cell Mol Biol.

[27] Doerr CH, Gajic O, Berrios JC, Caples S, Abdel M (2005). Hypercapnic acidosis impairs plasma membrane wound resealing in ventilator-injured lungs.. Am J Respir Crit Care Med.

[28] O'Toole D, Hassett P, Contreras M, Higgins BD, McKeown ST (2009). Hypercapnic acidosis attenuates pulmonary epithelial wound repair by an NF-kappaB dependent mechanism.. Thorax.

[29] Maccarrick MJ, Torbati D, Kimura D, Raszynski A, Zeng W (2010). Does hypercapnia ameliorate hyperoxia-induced lung injury in neonatal rats?. Lung.

[30] Masood A, Yi M, Lau M, Belcastro R, Shek S (2009). Therapeutic effects of hypercapnia on chronic lung injury and vascular remodeling in neonatal rats.. Am J Physiol Lung Cell Mol Physiol.

[31] Hudson LD, Milberg JA, Anardi D, Maunder RJ (1995). Clinical risks for development of the acute respiratory distress syndrome.. Am J Respir Crit Care Med.

[32] O'Croinin DF, Nichol AD, Hopkins N, Boylan J, O'Brien S (2008). Sustained hypercapnic acidosis during pulmonary infection increases bacterial load and worsens lung injury.. Crit Care Med.

[33] Chonghaile MN, Higgins BD, Costello JF, Laffey JG (2008). Hypercapnic acidosis attenuates severe acute bacterial pneumonia-induced lung injury by a neutrophil-independent mechanism.. Crit Care Med.

[34] Costello J, Higgins B, Contreras M, Chonghaile MN, Hassett P (2009). Hypercapnic acidosis attenuates shock and lung injury in early and prolonged systemic sepsis.. Crit Care Med.

[35] Carvalho CR, Barbas CS, Medeiros DM, Magaldi RB, Lorenzi FG (1997). Temporal hemodynamic effects of permissive hypercapnia associated with ideal PEEP in ARDS.. Am J Respir Crit Care Med.

[36] Galluccio ST, Rai S, Sharley P (2008). An unexpected ending: brain death following acute severe asthma.. Crit Care Resusc.

